# Prior oral contraceptive use in ovarian cancer patients: assessing associations with overall and progression-free survival

**DOI:** 10.1186/s12885-015-1774-z

**Published:** 2015-10-15

**Authors:** Aminah Jatoi, Nathan R. Foster, Kimberly R. Kalli, Robert A. Vierkant, Zhiying Zhang, Melissa C. Larson, Brooke Fridley, Ellen L. Goode

**Affiliations:** 1 Department of Oncology, Mayo Clinic, 200 First Street SW, Rochester, MN 55905 USA; 2 Department of Health Sciences Research, Mayo Clinic, Rochester, MN USA; 3 Department of Kinesiology and Community Health, University of Illinois at Urbana-Champaign, Champaign, IL USA; 4 Department of Biostatistics, University of Kansas Medical Center, Kansas City, KS USA

**Keywords:** Ovarian cancer, Survival, Oral contraceptives

## Abstract

**Background:**

Prior studies have described a reduced risk of developing ovarian cancer with the use of oral contraceptives. In this context, we decided to examine if oral contraceptive use prior to a diagnosis of ovarian cancer is associated with better overall and progression-free survival.

**Methods:**

This retrospective cohort study included ovarian cancer patients who were seen at the Mayo Clinic in Rochester, Minnesota from 2000 through 2013. Patients completed a risk factor questionnaire about previous oral contraceptive use, and clinical data were extracted from the electronic medical record.

**Results:**

A total of 1398 ovarian cancer patients responded to questions on oral contraceptive use; 571 reported no prior use with all others having responded affirmatively to oral contraceptive use. Univariate analyses found that oral contraceptive use (for example, ever versus never) was associated with better overall survival (hazard ratio (HR) 0.73 (95 % confidence interval (CI): 0.62, 0.86); p = 0.0002) and better progression-free survival (HR 0.71 (95 % CI: 0.61, 0.83); p < 0.0001). In multivariate analyses, contraceptive use continued to yield a favorable, statistically significant association with progression-free survival, but such was not the case with overall survival.

**Conclusions:**

This study suggests that previous oral contraceptive use is associated with improved progression-free survival in patients diagnosed with ovarian cancer.

Decades of data show that oral contraceptive use reduces the risk of ovarian cancer. A greater than 20 % relative risk reduction appears to occur for every 5 years a woman reports taking oral contraceptives [1]. This risk reduction is particularly salient among women who have used oral contraceptives for 10 years or longer at any point in their lives, and it also occurs in high-risk women, such as those with *BRCA1* and *BRCA2* germline mutations [2]. Continuous ovulation is thought to predispose to ovarian epithelial cell DNA damage, which in turn gives rise to carcinogenesis, thus providing mechanistic plausibility to how cessation of ovulation from oral contraceptives might lead to lower cancer risk [3]. Although large pooled analyses suggest that oral contraceptives could prevent 200,000 cases of ovarian cancer and 100,000 deaths from this malignancy over 20 years, such deductions have not spawned large-scale prevention trials [4, 5]. The many decades of follow up required to capture a small number of cancer cases, the enormous funding necessary to conduct prevention trials of sizable complexity, and the fact that oral contraceptives can also confer negative effects, such as an increased risk of thrombophlebitis and breast cancer, all lessen enthusiasm for the conduct of such prevention trials. Moreover, to date, the above robust observation has not yet dramatically changed clinical practice.

In contrast to these data on ovarian cancer prevention, few studies have specifically sought to assess whether oral contraceptives prior to an ovarian cancer diagnosis is associated with better outcomes after contracting this malignancy. This possibility builds on previous data on the purported role of oral contraceptives in preventing ovarian cancer. Moreover, in contrast to primary prevention, establishing this observation could lead to prospective research aimed at improving outcomes in ovarian cancer patients. Thus, to further examine the effects of previous oral contraceptives on outcomes in ovarian cancer patients, we studied patients at the Mayo Clinic in Rochester, Minnesota. Our main aim was to determine whether oral contraceptive use prior to a diagnosis of ovarian cancer is associated with better overall and progression-free survival within the context of in depth multivariate analyses undertaken within a consecutively-recruited and monitored cohort of ovarian cancer patients.

## Methods

### Overview

This study focused on women with invasive primary epithelial ovarian, fallopian tube, or peritoneal cancer seen at the Mayo Clinic in Rochester, Minnesota. The study of all these tumors in aggregate has substantial precedent because these malignancies behave and are treated similarly. The Mayo Clinic Institutional Review Board (IRB) approved this study. As described previously, patients were consecutively recruited from 2000 through 2013 from the Mayo Clinic in Rochester, Minnesota [6]. All patients had to be 20 years of age or older and had to have provided written informed consent. Patients then completed a paper risk factor questionnaire (see below) that included queries on previous oral contraceptive use. Trained medical personnel extracted details on tumor histology, type of surgery, and administration of chemotherapy from the electronic medical record.

### Study endpoints

Outcome data were acquired through April 2014. Data on cancer recurrence were updated via the Mayo Clinic electronic medical record and included a mailed questionnaire to patients and medical record review. Vital status was gleaned from the Mayo Clinic electronic medical record, the Mayo Clinic Cancer Registry, and registration records. Death certificates were requested from the appropriate government bodies with the appropriate permissions to confirm dates of death.

This study analyzed overall survival, as defined as the interval from a histologic-or cytologic-confirmed cancer diagnosis to date of death. If vital status was unknown for a specific patient, that patient was censored on the date of last contact or at five years, whichever occurred first. The rationale for this approach rests in the fact that the majority of ovarian cancer-related deaths occur in the first five years after diagnosis. Progression-free survival was also assessed and was defined as the date from cancer diagnosis to the date of initiation of second-line cancer treatment or death. Although vital status was assessed in all patients, progression-free survival had been assessed in only a subset.

### Definition of covariates

Oral contraceptive use was the main variable of interest, and it was assessed by means of a self-administered questionnaire. Patients were asked, “Have you ever used oral contraceptive pills (“the pill”)?” and were asked to mark the appropriate response of “yes” or “no.” If they answered “yes,” they were then asked to estimate duration of use in years, as summarized in this report as both a categorical variable (1–48 months and > 48 months) and a continuous variable. Other hormone-related variables were also assessed; these included age at menarche and menopause status. Patients were also assessed for number of live births, coded as nulliparous versus one or two versus three or more. This grouping of parity was done because of efforts to maintain statistical power and because it appeared clinically reasonable.

A variety of clinical covariates, many of which have prognostic associations, were also considered. These consisted of 1) cancer stage; 2) cancer histology: high grade serous, low grade serous, endometrioid versus clear cell, mucinous, mixed epithelial, borderline invasive mixed epithelial, and other; 3) tumor grade; 4) outcome of initial surgery: no residual disease versus </= 1 cm of residual disease versus > 1 cm residual disease; 5) platinum-based chemotherapy administered within the first three months of surgery: yes versus no [7]; 6) patient age at cancer diagnosis; 7) smoking history: never versus former versus current; and 8) first degree family history of breast or ovarian cancer: yes versus no.

### Analyses

Chi-square or Wilcoxon rank-sum tests were used, as appropriate, to compare all the covariates between never- and ever- oral contraceptive users. Univariate analyses were undertaken for all the variables described above. Oral contraceptive use was examined in two separate analyses: 1) based on a “ever” and “never” patient response and 2) based on duration of oral contraceptive use: never versus 1–48 months versus > 48 months or patient-reported years of use as a continuous variable. All variables were examined to assess their individual associations with overall survival and progression-free survival. Kaplan Meier curves were constructed to visualize unadjusted associations. Cox proportional hazards modeling accounting for left truncation was used for univariate and multivariate analyses with estimation of HRs and 95 % CIs. Left truncation is a standard method undertaken to limit sampling bias when one is unable to consistently observe the time when an event might have occurred.

Multivariate analyses were then conducted to identify the independent prognostic association of each of these variables and to estimate the effects of these variables on overall and progression-free survival endpoints. Three models were constructed with inclusion of 1) all variables except those with high rates of missing data; 2) variables that, in univariate analyses, had yielded a statistically significant association (p < 0.01) with overall and disease-free survival; and 3) variables that, in univariate analyses, had yielded a statistically significant association with overall survival and disease-free survival (p < 0.01) except those with notable missing data. These models were constructed in this manner to avoid biases that might arise from missing data. All statistical tests were two-tailed, and a p-value of < 0.05 is considered statistically significant. All statistical analyses were performed using Statistical Analysis Software version 9.3 (SAS Institute, Cary, North Carolina).

## Results

### Demographics

This study focused on 1398 ovarian cancer patients who had completed a questionnaire on oral contraceptive use at study entry. Within this cohort, 571 reported no prior oral contraceptive use. Among oral contraceptive users, the patient-reported median duration was 60 months (range: 1 to 444 months).

Baseline characteristics appear in Table 1. Patients who had used oral contraceptives were more likely to have had no residual disease from surgery but were less likely to have started platinum-based chemotherapy after surgery. Patients who had used oral contraceptives were also diagnosed at an earlier age and had fewer live births.

**Table 1:** Demographics

Characteristic	Oral contraceptive use^a^			*P*-value^b^
	Never-Used	Ever-Used	Total	
	*n* = 571	*n* = 827	*n* = 1398	
	(%)	(%)	(%)	
Age at diagnosis, median in years (range)	69 (24, 93)	58 (21, 91)	61 (21, 93)	<0.0001
Cancer stage				
1	77 (14)	149 (19)	226 (17)	0.07
2	34 (6)	44 (6)	78 (6)	
3	322 (60)	460 (59)	782 (60)	
4	106 (20)	124 (16)	230 (17)	
Tumor Type				
High-grade serous	320 (56)	419 (51)	739 (53)	0.09
Endometrioid	71 (12)	91 (11)	162 (12)	
Clear cell	32 (6)	46 (6)	78 (6)	
Mucinous	9 (2)	23 (3)	32 (2)	
Other	139 (24)	147 (29)	387 (27)	
Grade				
1	22 (5)	49 (8)	71 (7)	0.06
2	45 (10)	77 (12)	122 (11)	
3	260 (58)	362 (58)	622 (58)	
4	123 (27)	140 (22)	263 (24)	
Tumor Debulking Status				
No residual disease	218 (39)	393 (48)	611 (45)	0.004
Less than or equal to 1 centimeter	150 (27)	172 (21)	322 (24)	
Optimal but amount of residual disease unknown	79 (14)	105 (13)	184 (13)	
Suboptimal	79 (14)	86 (11)	165 (12)	
Unknown	33 (6)	57 (7)	90 (7)	
Platinum-based chemotherapy within 3 months of surgery				
Yes	309 (87)	530 (93)	839 (90)	0.002
No	48 (13)	42 (7)	90 (10)	
Age at menarche, median in years (range)	13 (9, 19)	13 (8, 19)	13 (8, 19)	0.19
Age at menopause, median in years (range)	50 (18, 76)	50 (21, 60)	50 (18, 76)	0.26
Number of live births				
0	113 (20)	139 (17)	252 (18)	<0.0001
1-2	177 (31)	374 (45)	551 (40)	
3 or more	278 (49)	313 (38)	591 (42)	
Smoking				
Never	342 (66)	480 (62)	822 (64)	0.25
Former	136 (26)	234 (30)	370 (29)	
current	38 (7)	63 (8)	101 (8)	
Breast or ovarian cancer in a first-degree relative				
Yes	133 (24)	188 (23)	321 (23)	0.75
No	426 (76)	628 (77)	1054 (77)	

^a^Numbers may not sum to the whole cohort or to 100 % either because of missing values or rounding, and numbers in parentheses represent percentages unless otherwise specified

^b^Chi square or Wilcoxon rank-sum test were used to compare Never- and Ever-Users, as appropriate

### Overall survival and progression-free

**Fig. 1:**
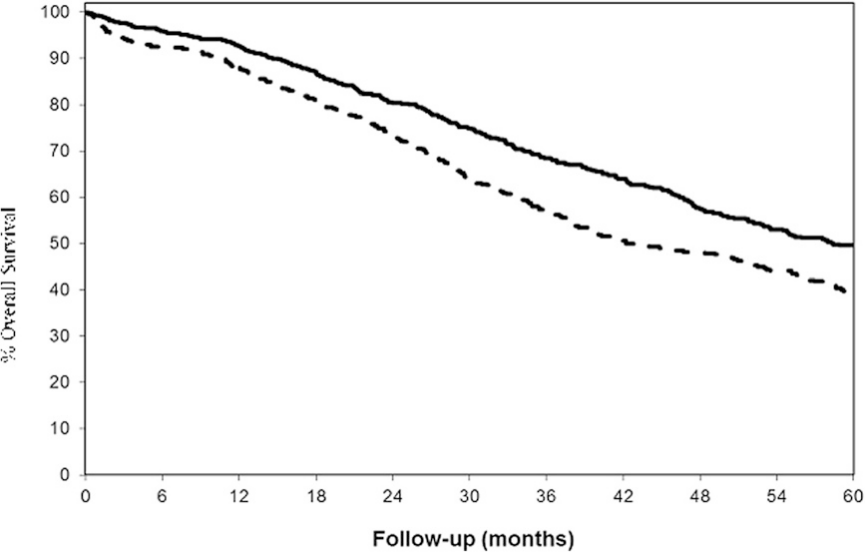
Overall Survival Based on Ever- (solid line) and Never-Users (dashed line) of Oral Contraceptives. In univariate analyses ever-users of oral contraceptives (*n* = 793) manifested a longer overall survival compared to never-users (dashed line) (*n* = 551) (HR = 0.73 (95 % CI: 0.62, 0.86); *p* = 0.0002 (accounting for left truncation). Within the cohort were a total of 562 deaths. Similar statistically significant findings were seen in univariate analyses when patients were dichotomized on the basis of duration of oral contraceptive use

**Fig. 2:**
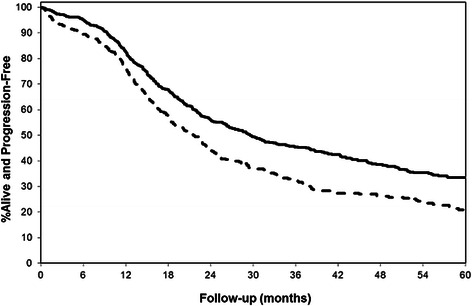
Progression-Free Survival Based on Ever- (solid line) and Never-Users (dashed line) of Oral Contraceptives. In univariate analyses ever-users of oral contraceptives (*n* = 700) manifested a longer progression-free survival compared to never-users (dashed line) (*n* = 489) (HR = 0.71 (95 % CI: 0.61, 0.83); *p* < 0.0001 (accounting for left truncation). Within the cohort were a total of 656 events of cancer progression. Similar statistically significant findings were seen in univariate analyses when patients were dichotomized on the basis of duration of oral contraceptive use

At the time of this report, 562 patients had died, and 656 had developed recurrent cancer or had died after accounting for left truncation. Univariate analyses, which do not take into account confounding factors, suggested that oral contraceptive use (ever versus never) was associated with better overall survival (HR 0.73 (95 % CI: 0.62, 0.86); p = 0.0002) (Fig. 1). Similarly, univariate analyses also suggest oral contraceptives (ever versus never) was associated with more favorable progression-free survival (hazard ratio (HR) 0.71 (95 % confidence interval (CI): 0.61, 0.83); p < 0.0001) (Fig. 2). These survival advantages were also observed when oral contraceptive use was further characterized based on duration of use. Compared to never users, patients who reported using oral contraceptive for one to 48 months manifested a more favorable overall survival and progression-free survival, as did patients who reported using them for more than 48 months (data not shown).

In the three constructed multivariate models, oral contraceptive use did not yield a statistically significant improvement in overall survival, but it did yield such an association with improved progression-free survival (Table 2).

**Table 2:** Multivariate analyses for overall survival and progression-free survival

Model	Oral contraceptive variable	N (events) for overall survival	Adjusted HR (95 % CI) for overall survival	*P*-Value	N (events) for progression-free survival	Adjusted HR (95 % CI) for progression – free survival	*P*-Value
All variables^a^	Ever vs Never Users	880 (393)	0.82 (0.66, 1.03)	0.09	821 (481)	0.78 (0.64, 0.96)	0.02
	Duration of use						
	1–48 months vs never	857 (383)	0.91 (0.70, 1.19)	0.27	800 (467)	0.71 (0.56, 0.91)	0.03
	>48 months vs never		0.80 (0.61, 1.05)			0.83 (0.66, 1.06)	
	Duration of use as a continuous variable	857 (383)	0.998 (0.996, 1.00)	0.06	800 (467)	0.999 (0.997, 1.001)	0.22
Only the Statistically Significant Univariate Variables^b^	Ever vs Never Users	950 (429)	0.89 (0.72, 1.10)	0.28	879 (520)	0.80 (0.66, 0.98)	0.03
	Duration of use						
	1–48 months vs never	926 (418)	1.01 (0.78, 1.30)	0.35	858 (506)	0.74 (0.59, 0.94)	0.04
	>48 months vs never		0.84 (0.65, 1.10)			0.86 (0.68, 1.08)	
	Duration of use as a continuous variable	926 (418)	0.998 (0.996, 1.000)	0.06	858 (506)	0.999 (0.997, 1.001)	0.17
Only the Statistically Significant Univariate Variables, Excluding Tumor Grade^c^	Ever vs Never Users	1204 (511)	0.95 (0.78, 1.15)	0.57	1111 (619)	0.84 (0.70, 0.996)	0.046
	Duration of use	1173 (497)		0.80	1083 (601)		0.052
	1–48 months vs never		1.00 (0.79, 1.27)			0.77 (0.62, 0.95)	
	>48 months vs never		0.93 (0.74, 1.18)			0.89 (0.72, 1.09)	
	Duration of use as a continuous variable	1173 (497)	0.999 (0.997, 1.001)	0.19	1083 (601)	0.999 (0.998, 1.000)	0.15

^a^Adjusted for all variables from Table 1 except for age at menarche, age at menopause, and platinum-based chemotherapy within 3 months, all of which were missing in approximately 450 patients

^b^Statistically significant variables in univariate analyses include tumor stage, tumor type, tumor grade, debulking status after surgery, age at cancer diagnosis, and number of live births

^c^Tumor grade was excluded because it was missing in approximately 300 patients

NOTE: When age was excluded, all the oral contraceptive use models reached statistical significance (*p* < 0.05), except in the progression-free survival model that used continuous duration of oral contraceptive use as the key variable

Of note, the multivariate models pointed to patient age as a major confounder, as younger age was strongly associated with oral contraceptive use. For example, in the first model, with no adjustment for age, oral contraceptive use was, in fact, associated with better overall survival (HR = 0.70; p < 0.001) as well as with better progression–free survival. However, after adjusting for age, this association with overall survival lost its statistical significance, although the association with improved progression-free survival was maintained. Furthermore, we performed separate analyses on associations with oral contraceptive use and overall survival and progression-free survival based on whether patients had residual disease postoperatively and found these prognostic associations with oral contraceptive use were sustained.

## Discussion

This study examined whether oral contraceptive use prior to a diagnosis of ovarian cancer was associated with improved overall survival and progression-free survival. We observed this protective association in univariate analyses, but multivariate analyses yielded less consistent findings. In the latter, prior oral contraceptive use was associated with improved progression-free survival but not with overall survival. Younger patients reported greater use of oral contraceptives as well as longer survival. This study provides corroborative evidence that previous oral contraceptive use is associated with better clinical outcomes in patients diagnosed with ovarian cancer, at least with respect to progression-free survival.

Indeed, our findings are particularly noteworthy because of the detailed nature of our multivariate analyses. The fact that we were able to adjust for highly relevant clinical co-variates such as the extent of the primary debulking surgery and the fact that we had detailed follow up information on consecutively-treated patients strengthen this report. Our study provides an important contribution to an emerging body of literature that indicates oral contraceptive use prior to a diagnosis of ovarian cancer is associated with better outcomes. Only a few studies have examined whether oral contraceptives appear to change outcomes in patients who develop ovarian cancer at a later date. First, using the Nurses’ Health Study, the New England Case–control Study, the Australian Ovarian Cancer Study, and the NIH-AARP Diet and Health Study, Poole and others examined numerous lifestyle factors and their effect on clinical outcomes in ovarian cancer patients [5]. Among 4,342 patients with ovarian cancer, previous oral contraceptive use was associated with a lower risk of death (five-year increase in relative risk 0.69 (95 % confidence interval (CI): 0.58, 0.82)). These investigators noted that their study design might not have captured patients with rapidly fatal malignancies and that limited clinical data were available to accommodate some of their analyses. Nonetheless, this observation appears plausible, particularly given the earlier-referenced studies that have focused on cancer prevention. Second, several investigators, including Vessey and others from the Oxford Family Planning Association Contraceptive Study, Hannaford and others from the Royal College of General Practitioners’ Oral Contraceptive study, and those from the Collaborative Group on Epidemiological Studies of Ovarian Cancer have also reported decreased overall mortality among ovarian cancer patients who had used oral contraceptives prior to their cancer diagnosis [1, 8, 9]. The above two studies used a cohort design of oral contraceptive users and non-users and reported on death from ovarian cancer. These studies confirm the observation from Poole and others, although their primary goal was to understand cancer risk.

However, not all studies of oral contraceptive use and outcome have been consistent. For example, Nagle and others reported on 676 women diagnosed with ovarian cancer, and, although 310 women had used oral contraceptives, the latter did not demonstrate a protective association with respect to ovarian cancer mortality (adjusted hazard ratio (HR) 0.88 (95 % CI: 0.70, 1.11) [10]. This study examined a cohort of women with ovarian cancer and looked at survival of cancer patients who were users of oral contraceptives and cancer patients who were not users of oral contraceptives. These authors concluded that “reproductive and hormonal exposures prior to diagnosis do not influence survival from invasive ovarian cancer, in contrast to their substantial effects on the etiology of this disease,” and others have drawn similar conclusions [11]. Taken together, these studies provide justification for generating the study reported here.

Is this favorable association between prior oral contraceptive use and survival mechanistically plausible? It appears to be. First, as alluded to earlier, previous studies that have shown oral contraceptives protect against the development of primary ovarian cancer suggest that cessation of ovulation halts the repeated monthly trauma that occurs on the surface of the ovary, thereby limiting the possibility of epithelial cell mutation and subsequent carcinogenesis [3]. Similar mechanisms might be invoked to explain the favorable prognostic associations observed here. In an analogous fashion, epithelial ovarian cancer cells that undergo repeated, monthly trauma from ovulation are perhaps more likely to develop DNA mutations. The more frequent the trauma, the more apt these cells are to develop aberrant DNA mutations; the more numerous the DNA mutations, the more aggressive the cancer [12]. Although this line of thinking may contradict the hypothesis that ovarian cancer originates from fallopian tube fimbria, it nonetheless merits consideration, particularly because the fimbria are also exposed to hormones in the follicular fluid [13]. Second, in a preclinical model, Romero and others observed that contraceptive hormone exposure decreased matrix metalloproteinase-2 activity, invoking this observation to explain the effects of oral contraceptives on carcinogenesis and perhaps also on the improved clinical outcomes observed by us and others [14]. One might speculate that the role of matrix metalloproteinase-2 proteins in modifying the extracellular matrix confers long-term consequences that attenuate the malignant potential of ovarian cancers and provide greater susceptibility to cancer treatment. In view of a growing literature that underscores an inverse association between oral contraceptive use and poor outcomes from ovarian cancer, it appears important to probe into and delineate the mechanisms that underlie these observations, such as those posited above.

Our study has at least three limitations. First, the questionnaire we used did not capture detailed information on oral contraceptive product formulation, which may be informative, as oral contraceptives with high progesterone content appear to carry a more protective effect [15]. Second, the exact cause of death for many patients is still being curated and thus cause-specific mortality was not analyzed here, although our use of censoring data at date of last contact and limiting follow up to 5 years post-diagnosis are attempts to mitigate this limitation. Nonetheless, it remains possible that deceased older patients had died more frequently of non-cancer causes, a plausible scenario that might explain why our study did not reveal an improvement in overall survival with oral contraceptive use in multivariate analyses, despite having captured an improvement in progression-free survival. Finally, this study provides limited data on how recently oral contraceptives had been used, and such timing issues would likely have an important impact on the strength of this association. Despite such limitations, our study -- coupled with several that preceded it -- points to a need to investigate mechanisms that explain how and why prior oral contraceptive use appears to improve clinical outcomes in ovarian cancer patients. Understanding such mechanisms might lead to more effective therapeutic interventions in patients diagnosed with this malignancy.

## References

[1] Beral V, Doll R, Hermon C, Peto R, Reeves G, Collaborative Group on Epidemiological Studies of Ovarian Cancer (2008). Ovarian cancer and oral contraceptives: collaborative reanalysis of data from 45 epidemiological studies including 23, 257 women with ovarian cancer and 87,303 controls. Lancet.

[2] Friebel TM, Domcheck SM, Rebbeck TR (2014). Modifiers of cancer risk in BRCA1 and BRCA2 mutation carriers: systemic review and meta-analysis. J Nat Cancer Inst.

[3] Wong AS, Leung PC (2007). Role of endocrine and growth factors on the ovarian surface epithelium. J Obstet Gyneacol Res.

[4] Bosetti C, Negri E, Trichopoulos D, Franceschi S, Beral V, Tzonou A (2002). Long-term effects of oral contraceptives on ovarian cancer risk. Int J Cancer.

[5] Poole EM, Merritt MA, Jordan SJ, Yang HP, Hankinson SE, Park Y (2013). Hormonal and reproductive risk factors for epithelial ovarian cancer by tumor aggressiveness. Cancer Epidemiol Biomarkers Prev.

[6] Peethambaram P, Fridley BL, Vierkant RA (2011). Polymorphisms in ABCB1 and ERCC2 associated with ovarian cancer outcome. Int J Mol Epidemiol Genet.

[7] Hofstetter G, Concin N, Braicu I, Chekerov R, Sehouli J, Cadron I (2013). The time interval from surgery to start of chemotherapy significantly impacts prognosis in patients with advanced serous ovarian carcinoma – analysis of patient data in the prospective OVCAD study. Gynecol Oncol.

[8] Vessey M, Yeates D (2013). Oral contraceptive use and cancer: final report from the Oxford-Family Planning Association contraceptive study. Contraception.

[9] Hannaford PC, Selvaraj S, Elliott AM, Angus V, Iversen L, Lee AJ (2010). Cancer risk among users of oral contraceptives: cohort data from the Royal College of General Practitioner’s oral contraceptive study. BMJ.

[10] Nagle CM, Bain CJ, Green AC, Webb PM (2008). The influence of reproductive and hormonal factors on ovarian cancer survival. Int J Gynecol Cancer.

[11] Yang L, Klint A, Lambe M, Bellocco R, Riman T, Bergfeldt K (2008). Predictors of ovarian cancer survival: a population-based prospective study in Sweden. Int J Cancer.

[12] Giam M, Rancati G. Aneuploidy and chromosomal instability in cancer: a jackpot to chaos. Cell Div. 2015;20. 10.1186/s13008-015-0009-7 PMC4443636 26015801

[13] Emori MM, Drapkin R. The hormonal composition of follicular fluid and its implications for ovarian cancer pathogenesis. Reprod Biol Endocrinol. 2014;12:60. 10.1186/1477-7827-12-60 PMC4105128 24997727

[14] Romero IL, Gordon IO, Jagadeeswaran S, Mui KL, Lee WS, Dinulescu DM (2009). Effects of oral contraceptives or gonadotropic-releasing hormone agonist on ovarian carcinogenesis in genetically engineered mice. Cancer Prev Res.

[15] Schildkraut JM, Calingaert B, Marchbanks PA, Moorman PG, Rodriguez GC (2002). Impact of progestin and estrogen potency in oral contraceptives on ovarian cancer risk. J Natl Cancer Inst.

